# Five years after Treat All implementation: Botswana’s HIV response and future directions in the era of COVID-19

**DOI:** 10.4102/sajhivmed.v22i1.1275

**Published:** 2021-10-15

**Authors:** Keith Jefferis, Ava Avalos, Heston Phillips, Mpho Mmelesi, Dinah Ramaabya, Bornapate Nkomo, Charles Muthoga, Joseph N. Jarvis, Siphiwe Ratladi, Robert Selato, John Stover

**Affiliations:** 1 E-consult Botswana, Gaborone, Botswana; 2 Careena Centre for Health, Gaborone, Botswana; 3 UNAIDS, Lusaka, Zambia; 4 UNAIDS, Gaborone, Botswana; 5 Botswana Ministry of Health and Wellness, Gaborone, Botswana; 6 Botswana Harvard AIDS Institute Partnership, Gaborone, Botswana; 7 Department of Clinical Research, Faculty of Infectious and Tropical Diseases, London School of Hygiene and Tropical Medicine, London, United Kingdom; 8 National AIDS and Public Health Agency, Gaborone, Botswana; 9 Avenir Health, Glastonbury, United States of America

**Keywords:** Botswana, Spectrum, GOALS, economic modelling, COVID-19, treat all

## Abstract

**Background:**

As the relentless coronavirus disease-2019 (COVID-19) pandemic continues to spread across Africa, Botswana could face challenges maintaining the pathway towards control of its HIV epidemic.

**Objective:**

Utilising the Spectrum GOALS module (GOALS-2021), the 5-year outcomes from the implementation of the Treat All strategy were analysed and compared with the original 2016 Investment Case (2016-IC) projections. Future impact of adopting the new Joint United Nations Programme on HIV/AIDS (UNAIDS) Global AIDS Strategy (2021–2026) targets and macroeconomic analysis estimating how the financial constraints from the COVID-19 pandemic could impact the available resources for Botswana’s National HIV Response through 2030 were also considered.

**Method:**

Programmatic costs, population demographics, prevention and treatment outputs were determined. Previous 2016-IC data were uploaded for comparison, and inputs for the GOALS, AIM, DemProj, Resource Needs and Family Planning modules were derived from published reports, strategic plans, programmatic data and expert opinion. The economic projections were recalibrated with consideration of the impact of the COVID-19 pandemic.

**Results:**

Decreases in HIV infections, incidence and mortality rates were achieved. Increases in laboratory costs were offset by estimated decreases in the population of people living with HIV (PLWH). Moving forward, young women and others at high risk must be targeted in HIV prevention efforts, as Botswana transitions from a generalised to a more concentrated epidemic.

**Conclusion:**

The Treat All strategy contributed positively to decreases in new HIV infections, mortality and costs. If significant improvements in differentiated service delivery, increases in human resources and HIV prevention can be realised, Botswana could become one of the first countries with a previously high-burdened generalised HIV epidemic to gain epidemic control, despite the demands of the COVID-19 pandemic.

## Introduction

Botswana has made substantial gains against the HIV epidemic and is one of the few countries in Africa to have reached the Joint United Nations Programme on HIV/AIDS (UNAIDS) 90-90-90 targets.^1^ It was also one of the first African countries to successfully implement a Treat All strategy, which included antiretroviral (ART) treatment optimisation using the integrase inhibitor dolutegravir, following extensive programmatic and economic modelling contained within the 2016 Investment Case (2016-IC).^2^ This strategic investment was aimed at reinvigorating the country’s National HIV Response and safeguarding the gains already made against the HIV epidemic. For more than 20 years, the Government of Botswana had financed the largest portion of its National HIV Response, contributing more than 65% of its total HIV expenditures.^2^ Together with generous donor and development partner funding as well as a progressive government-led multisectoral approach, the country was able to decrease infections, save lives and approach epidemiologic control of HIV.

As the global coronavirus disease-2019 (COVID-19) pandemic, however, continues to negatively impact the country’s medical infrastructure and its global economic standing, Botswana may face serious challenges to maintain its current level of healthcare capacity and control of its HIV epidemic. It, therefore, remains critical to clearly delineate and prioritise the areas of HIV prevention, treatment and care that will have the greatest impact to ensure economic and programmatic sustainability over the next decade. To this end, in cooperation with the Ministry of Health and Wellness and National AIDS and Health Promotion Agency, a technical working group composed of programmatic, economic, clinical and modelling experts used the GOALS module of Spectrum (GOALS-2021) to analyse the 5-year outcomes since the implementation of the Treat All strategy in 2016. The outcomes are then compared with the original 2016-IC projections. The work also considers the future programmatic and economic impact of adopting the new UNAIDS Global AIDS Strategy targets through 2030 to end HIV as a public health threat. Additional macroeconomic analysis estimates how the financial constraints resulting from the COVID-19 pandemic might impact the available resources required to maintain the country’s National HIV Response. Whilst it was projected that the adoption of the Treat All Strategy and the use of dolutegravir would reduce new HIV infections, HIV mortality and overall costs, this was the first analysis since 2016 that aimed to quantify and test these estimations.

## Methods

### Modelling analysis

Spectrum is a modelling system used by HIV experts and policymakers as an analytical tool to support the decision-making processes. The model is designed to use the available country-level data within specific modules to produce outputs that are relevant to programme policy and planning, and the software is continuously updated. Using Spectrum version 6.06 (2021),^3^ programmatic costs, as well as demographic, prevention and treatment-related outputs, were determined.

The original 2016-IC data were also uploaded into Version 6.06 for comparison. Prevention data and revised inputs were then used to produce the GOALS-2021 module through 2030. Inputs for the Resource Needs and Family Planning modules of Spectrum were derived from published reports, strategic plans, programmatic data and expert opinion (See Appendix 1: Inputs and targets by 2030 for the 2016-IC, AIM-2020, GOALS-2021 and the UNAIDS Global AIDS Strategy and Appendix 3: Data sources for Spectrum).

Additional Spectrum modules used for this analysis included DEMPROJ for demographic characteristics of the population by age and sex, including assumptions on fertility, mortality and migration; AIM to estimate the consequences of the HIV epidemic, such as the number of people living with HIV (PLWH), new HIV infections, and AIDS deaths; FAMPLAN to determine the family planning requirements to reach national goals; GOALS to estimate the costs and impact of the HIV interventions; and Resource Needs to estimate resources needed for the implementation of the HIV programme, including the cost of care and treatment, prevention, and policy and programmes.

### Economic analysis

The 2016-IC whilst published in 2016 was prepared in 2015 and was based mainly on economic and fiscal data up to 2014, with projections of the period up to 2030. In 2021, the 6-year period from 2015 to 2020 was evaluated based on actual data. Economic projections were recalibrated with consideration of the possible impact of the COVID-19 pandemic, as well as the updated GOALS-2021 cost projections. Currency conversions were made at $1.00 to 11.2 Botswana Pula (BWP), consistent with the conversions used in the 2016-IC. The following assumptions were also made:

- Donor funding is reduced by 5% a year in real USD terms from the 2016 level (BWP 550 million; $51 million), resulting in the contribution being reduced by 50% in real terms to $25m (in 2016 prices) by 2030.
- Private funding (through direct corporate spending and medical aid schemes) will remain constant at 10% of total national treatment costs through to 2030.
- Public spending is sufficient to fill the gap between the donor and private funding and total treatment and programme costs.
- The COVID-19 pandemic is well controlled without catastrophic economic or human resource demands.

## Modelling results

### Total HIV population

Comparisons of the GOALS-2021 outputs for the total HIV population were made using the UNAIDS Global AIDS Strategy outputs. By adopting the new UNAIDS strategy, Botswana could see a decline in the HIV population of approximately 41 500 over the period 2021–2030 because of reductions in HIV infections. However, without implementation, it is unlikely that there would be substantial decreases overall in the HIV population through 2030.

GOALS-2021 estimated the total HIV population for Botswana at 375 900 (353 520 – 400 150) in 2020. It is important to note, however, that from routine adjustments of the Spectrum model and the addition of actual programmatic data over the last 5 years (2015–2020), the estimates of the overall HIV population are lower by 44 404 cases from the original 2016-IC model estimates.

### New HIV infections

The estimates of annual new HIV infections that were projected in the 2016-IC are closely aligned with the GOALS-2021 projections, with a predicted decrease in annual newly acquired HIV infections from 9067 in 2016 to approximately 4406 by 2030. By implementation of the UNAIDS Global AIDS Strategy, there would be predictably further reduction to 2772 new infections by 2030.

There are two important points to note: firstly, although the 2021 National Spectrum AIM Model projects 8568 annual new HIV infections by 2021, which is significantly higher than the GOALS-2021 estimates at 4640, the AIM Module does not consider how all prevention and behavioural interventions impact these projections.^4^ Secondly, the Botswana AIDS Incidence Survey (BAIS) is a household epidemiological survey that should be completed every 4 years so that all the Spectrum projections can be aligned with the results of the actual country incidence survey. The last BAIS survey was carried out in Botswana in 2012. Until the updated results are made available, there remains some degree of uncertainty with all Botswana HIV modelling projections. It is expected that the results of BAIS-V, currently underway, will be available in early 2022, at which time incidence projections can be validated.

The larger decreases in HIV infections demonstrated using the new targets of the UNAIDS Global AIDS Strategy suggests that even further decreases in new HIV infections could be realised by expanding prevention interventions, such as broadly increasing access to pre-exposure prophylaxis (PrEP) across all high-risk populations including men who have sex with men (MSM), female sex workers (FSWs) and those who participate in transactional sexual encounters.

### HIV incidence

**FIGURE 1:**
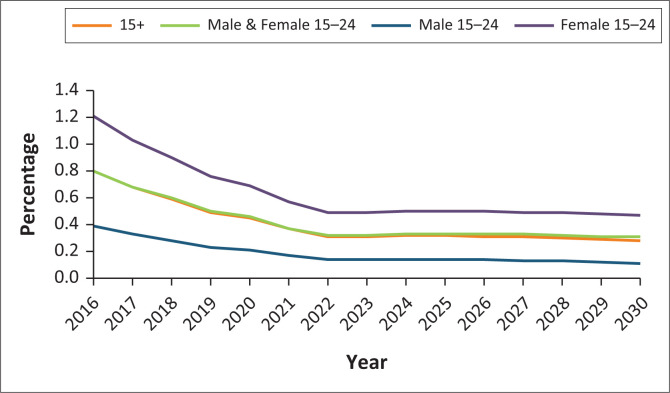
Incidence amongst young people aged 15–24 and adults aged 15+.

As concluded in the 2016-IC, young women remain at the highest risk of HIV acquisition (Figure 1). At the current prevention levels, young women will continue to have greater than four times the incidence of young men by 2030 and more than double the incidence of adults overall. This indicates that unless even more resources are invested into programmes targeting young women, their HIV infection rates will continue at the current levels without improvement, and they will continue to suffer the burden of HIV disproportionately.

Although the incidence rates of HIV have fallen steadily since 2016 when the Treat All Strategy was first launched, the continued higher levels of HIV infections reported amongst young women demonstrate that there is a transition of HIV epidemic in Botswana from being a generalised epidemic to one that requires a more targeted and differentiated approach towards high-risk populations. This is particularly true for young women who continue to experience higher levels of unemployment, further exacerbating vulnerability to transactional sexual encounters and other high-risk sexual behaviours.^5^

**FIGURE 2:**
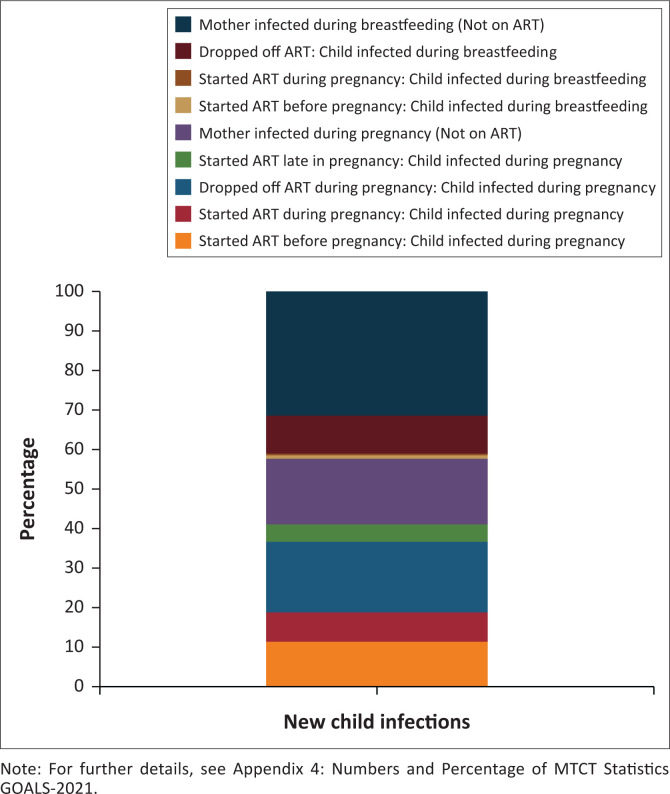
Source of new child infections.

Figure 2 highlights the need for also targeting high-risk women of child-bearing age to prevent mother-to-child transmission of HIV, with the overall transmission rate in Botswana for 2020 being less than 2%, one of the lowest estimated globally.^4^ There were 229 infections estimated in 2020, 9.6% because of mothers who dropped off antiretroviral treatment (ART) during pregnancy and 42.4% of all childhood infections occurring during the breastfeeding period.^6^ The results from GOALS-2021 reveal that Sexual Reproductive Health (SRH) interventions such as the expansion of the contraceptive method mix and training in its delivery, as well as providing PrEP during pregnancy and breastfeeding to high-risk women, are now critical to reverse these trends and decrease the incidence of HIV during pregnancy.

Although the Spectrum model does not estimate the prevalence of gender-based violence (GBV), its rise in Botswana is well documented with estimates as high as double the global average.^7^ Increases in GBV during the lockdown as a result of the COVID-19 pandemic, and the lack of contraception and shelter for abused women and children continue to put women at greater risk of HIV infection.^8,9^

### HIV Mortality

**FIGURE 3:**
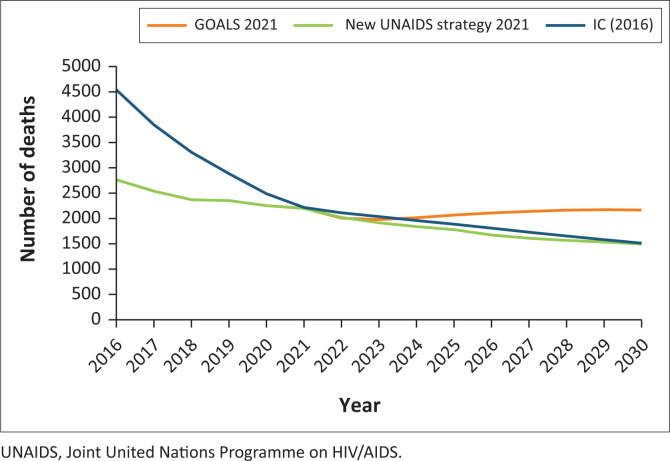
Annual AIDS deaths: 2016–2030.

For the past decade, HIV continues to be the number 1 cause of death in Botswana.^10^ Figure 3 demonstrates that gains against HIV mortality could be made by expanding prevention interventions, as well as improving the management of advanced HIV cases and comorbidities. Both the UNAIDS Global AIDS Strategy and the 2016-IC models incorporate higher HIV prevention targets than what is currently being implemented in Botswana. Reinvigorating Botswana’s HIV prevention programmes will not only prevent HIV infections but also save hundreds more lives.

Further decreases in rates of HIV mortality would be achieved through continued efforts to expand HIV testing availability, including home testing and linkage to care. Expanding the clinical capacity of healthcare workers to manage advanced HIV disease and comorbidities must also be prioritised. Studies conducted in 2020 reported that out of 14 423 newly initiated patients on ART in urban Botswana, 25% presented for ART initiation with CD4 counts < 200 cells/mL.^11^ As the average age of patients on ART continues to rise, managing serious comorbidities and non-communicable diseases will also increase the risks of clinical complications and drug-drug interaction from polypharmacy.

A brief report published in the *Journal of Clinical Infectious Disease* in 2020 shared results from a cryptococcal antigen study conducted in 2018–2019, which revealed that 76% of patients identified with CD4 counts < 200 cells/mL were already ART experienced, highlighting the importance of tracking patients who default or are lost to follow-up and improving adherence and psychosocial interventions.^12^

Deaths from cancers also continue to rise within the HIV population in Botswana. According to the National Cancer Registry in 2015, 61.6% of all cancer patients were infected with HIV.^13^ In 2020, tuberculosis became the fifth highest cause of mortality in 2009 and the seventh highest cause of mortality by 2019.^10^ According to the WHO, HIV represented 53.8% of all tuberculosis cases in 2018.^14^ Whilst the incidence of tuberculosis is reducing, it remains a serious risk for PLWH.

### Epidemic transition

**FIGURE 4:**
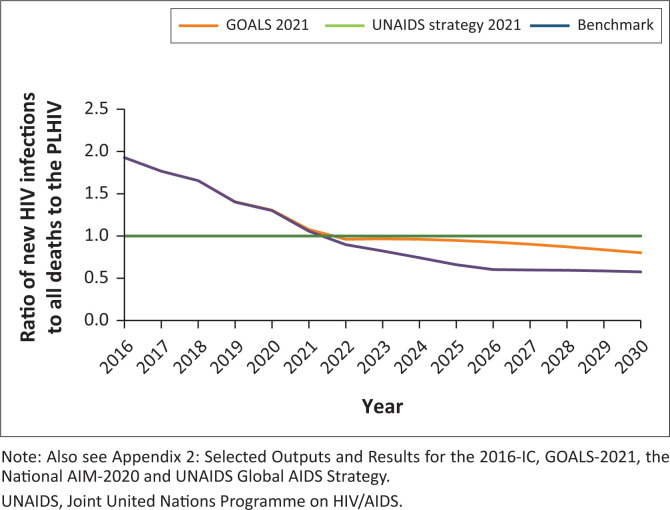
Incidence mortality ratio.

Until the results of BAIS V-2021 are complete, it will remain uncertain whether Botswana is trending towards or has already achieved epidemiologic transition, defined as when a country is on a trajectory to control the epidemic. Remarkably, all the three models predict that epidemic transition may occur as early as the end of 2022 (Figure 4). Although promising, this should not cause complacency within the National HIV response but rather highlight the urgent need for continuing to initiate more effective prevention interventions to decrease infections and deaths overall, and better safeguard the HIV population in the era of COVID-19, and global political and economic uncertainty.

### Economic analysis

#### GDP

Economic growth has been slightly higher than expected since the completion of the 2016-IC, with total GDP growth of 19.2% between 2015 and 2019, compared with the 17.3% projected in 2015. However, the most dramatic impact has been observed in 2020 as a result of COVID-19, leading to a GDP contraction of almost 9%, compared with the expected growth of around 4%. As a result, real GDP in 2020 was 11% lower than anticipated at the time of preparation of the 2016-IC. Much of this loss will be permanent, and although there will be some recovery of GDP lost in 2020, it is projected that real GDP in 2030 will now be 6% smaller than what was projected when the 2016-IC was prepared.

#### Government budget

**FIGURE 5:**
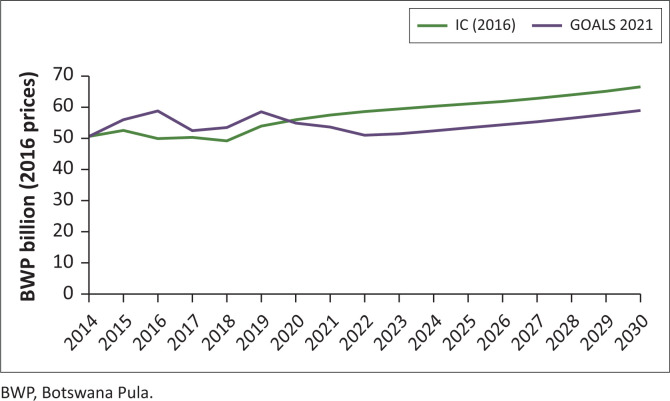
Real government spending (total).

The government budget also followed a different course to that anticipated in the 2016-IC (Figure 5). It was assumed then that there would be consistent efforts to contain spending and reduce budget deficits given the anticipated decline in revenues. In fact, spending has been higher than anticipated and budget deficits larger, compounded by the impact of COVID-19. In the medium term, the smaller size of the economy will have implications for the availability of funds to meet the needs of public spending across the board. The long-term need to contain spending in line with the anticipated decline in revenues remains. By 2030, total government spending is projected to be 11% lower than what was estimated in the 2016-IC.

#### Fiscal resources for HIV-AIDS spending

Fortunately, the lower projected costs for HIV spending under the GOALS-2021 scenario appears to be manageable even within the reduced overall envelope for public spending. The requirements for public spending on HIV depend on projections of donor funding and private healthcare spending. Even with the assumptions outlined in the methodology of decreased donor funding by 50% in real terms and maintaining the private funding at 10% of total national treatment costs by 2030, public spending is sufficient to fill the gap between the donor and private funding and total treatment and programme costs.

**FIGURE 6:**
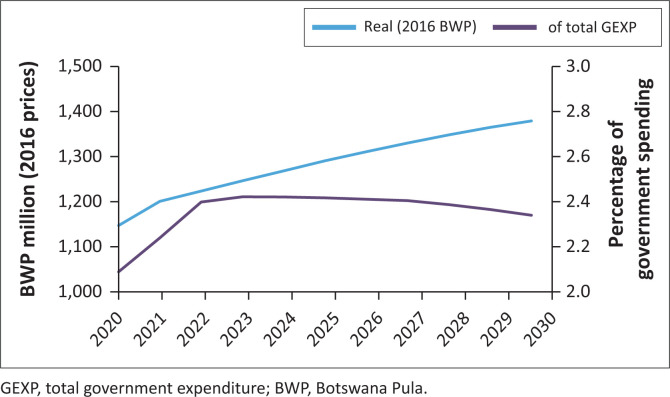
Public spending on HIV.

The outcome is that public spending on HIV will need to increase modestly from P1.15 billion (in constant 2016 BWP prices) in 2020 to P1.38bn in 2030. As a proportion of total public spending, the requirement will increase from an estimated 2.1% in 2020 to 2.4% in 2023 before declining slightly to 2.3% by 2030. This is lower than the estimated P1.66bn (2.8% of total spending) that would have been required to fully fund HIV spending needs in 2016 (Figure 6). If, however, public spending on HIV does not increase as indicated and is maintained at the same proportion of overall public spending as in 2020, then a financing gap of up to P235m (2016 prices) is projected, which would have to be filled by donor funding if total HIV spending is to be maintained.

It is important to note that costs of first-line ART regimens fell substantially since the implementation of the Treat All strategy in 2016.^15^ The estimated future costs for ART regimens contained within the IC-2016 were set before the global generic costs for dolutegravir had been established.^16^ Anticipating a significant cost reduction, the price of ART was set at 50% less beginning in 2020. In fact, the cost of generic co-formulated tenofovir-3TC-dolutegravir (TLD) fell to levels that were 66% less than the original 2016-IC projections.^15^

Costs of laboratory commodities and reagents that were used in the 2016-IC, however, significantly increased.^17^ This may be the result of a more comprehensive costing that was completed in 2021.^18^ Whilst it is highly unlikely that global prices for ART will decline any further, advocacy for reductions in laboratory reagents and commodities should be taken up on the national and global level with the same ferocity as was seen for the reduction in ART treatment costs and current demands for global COVID-19 vaccine equity. This advocacy is now taking place by necessity as low- and middle-income countries demand international patent waivers for COVID-19 vaccine technologies and other essential medications, including access to affordable contraception.

## Discussion

Botswana’s progressive National HIV Response has continued to optimise the delivery of ART treatment and care, as evidenced with the adoption of the Treat All strategy and the first use of dolutegravir in 2016. Five years later, more than 97% of patients on ART have achieved and maintained viral suppression,^19^ and the incidence of HIV is expected to have dropped to less than 1%.^4^ With low overall HIV positive testing yields and estimated ART coverage of 95% projected in GOALS-2021 by 2030, it appears that there is a transition of HIV epidemic in the country from a generalised epidemic to one that is concentrated within specific geographical locations and amongst the most vulnerable and high-risk populations.^20^ The results of the BAIS V-2021, expected in early 2022, will confirm whether these modelling predictions hold. Nonetheless, it is likely that results will fall somewhere between the National Model (AIM-2020) and GOALS-2021 Model estimates (see Appendix 2: Selected Outputs and Results by 2030 for the 2016-IC, AIM-2020, GOALS-2021 and UNAIDS Global AIDS Strategy). These projections are encouraging, as all models show significant reductions in key HIV response indicators.

Economic estimates predict that if the Government of Botswana can overcome the substantial economic hardships and human resource constraints caused by the COVID-19 pandemic, it should be able to continue to finance the greatest share of the financial requirements of its National HIV Response through 2030 – even at the current level of treatment costs and before optimisation of laboratory expenses – if the current level of public spending follows the national economic expectations. However, in order to ensure further reductions in HIV infections and cost savings, Botswana must focus its efforts on more targeted interventions for maximal impact. Priority areas should include at a minimum the following:

- Greater investment in SRH programming for young women of child-bearing age. This includes improved implementation of the availability of contraception, PrEP for high-risk pregnant and breastfeeding women, larger investment in economic opportunities for young women, and substantial investment in the reduction of maternal mortality and GBV that has continued to rise as a result of the COVID-19 pandemic.
- With low levels of HIV transmission and high coverage of ART, targeted HIV prevention interventions should continue to be rapidly and broadly expanded for those at highest risk for HIV infection. Expanded community interventions should also be prioritised for districts with the highest incidence rates and for geographically hard-to-reach populations, as well as those who may avoid public healthcare facilities, such as MSM, FSW, and those who engage in transactional sexual encounters. Additionally, detailed costing and efficiency gains studies of targeted ART-based prevention programmes should be carried out.
- Action and advocacy at the national and international level for cost reductions in laboratory reagents, commodities and supplies should be made a strategic priority. Laboratory expenses are now more than three times the cost of ART in Botswana.^18^ Therefore, further laboratory costing and cost-efficiency studies are required as a matter of urgency. Additional laboratory saving would also be realised if the Botswana National ART Treatment Guidelines were revised to decrease laboratory requirements for long-standing virally suppressed and treatment adherent PLWH, who might need less frequent monitoring.
- Investment in the establishment of differentiated care for people living with advanced HIV disease and streamlined service delivery for stable patients would substantially decrease opportunity costs for patients and provide much needed relief at all levels for health workers involved in HIV care and treatment. Financial investment in medical service delivery innovation within primary care for PLWH would likely show solid financial and human resource returns for the government and patients alike.
- Further investment in capacitating healthcare workers to competently manage advanced HIV care patients should also be prioritised. This is particularly important as the complications and comorbidities that will arise from acute and long-term COVID-19 infection emerge. Additional investments in COVID-19 and HIV co-infection surveillance and clinical research will also prove essential as the medical and economic aftermath of the pandemic becomes known.

## Conclusion

The implementation of the Botswana Treat All strategy in 2016 reinvigorated the country’s HIV response and contributed positively to decreases in HIV infections, mortality and costs, based upon modelling results and economic analysis completed 5 years after the strategy was launched. Costing estimates made in the IC-2016 also proved to be accurate, despite significant increases in laboratory expenses, which were offset by the lowered estimates of the overall HIV population.

With continued widespread access to HIV testing, ART treatment and care, Botswana is likely to achieve the UNAIDS 95-95-95 before the year 2025, if targeted HIV prevention interventions can be sustained and successfully implemented across all high-risk populations. If the final incidence results of the BAIS V-2021 Survey prove to be aligned with the GOALS-2021 Spectrum estimates and if significant improvements in differentiated service delivery can be realised, Botswana could become one of the first countries with a previously high-burden, generalised HIV epidemic to transition to epidemic control. Importantly, as a result of the country’s progressive financial investment and the continued optimisation of ART treatment, along with the dramatic decline in the costs of ART, the HIV epidemic in Botswana is no longer the major driver of health costs overall.

Moving forward, by ring fencing approximately 2.4% of GDP, in addition to continued donor and private funding, the Government of Botswana should be able to financially maintain its current HIV response. However, if the health demands of the country’s COVID-19 pandemic are not controlled successfully and the country’s economic recovery falls short, this could, instead, negatively impact the success of the country’s HIV epidemic control. Therefore, the economic impact of COVID-19 must continue to be closely monitored, and the commitment towards ending Botswana’s HIV epidemic must be further strengthened.

## Appendix 1

**TABLE 1-A1:** Inputs and targets by 2030 for the 2016-IC, GOALS 2021 and UNAIDS global AIDS strategy.

MODELS	2016 (%)	2020	2025	2030
**ART coverage (Adults 15+ years)**				
IC2016	75	85	92	95
GOALS 2021	75	87	93	95
UNAIDS	75	87	95	95
**PrEP coverage (MSM & FSW)**				
IC2016	MSM & FSW: 0	MSM & FSW: 1.0	MSM & FSW: 20	MSM & FSW: 20
GOALS 2021	MSM & FSW: 0	MSM & FSW: 2.0	MSM & FSW: 6	MSM & FSW: 10
UNAIDS	MSM & FSW: 0	MSM: 6% FSW: 9	MSM: 28 FSW: 45	MSM: 50 FSW: 80
**SRH family planning needs met**				
IC2016	64	69	74	80
GOALS 2021	63.5	6767	70	75
UNAIDS	63.5	-	80	90
**Condon coverage**				
IC2016 (General Population)	67	77	84	90
GOALS 2021	67	72	83	90
UNAIDS	67	72	83	90
**SMC**				
IC2016	48	60	70	80
GOALS 2021	35	46	60	77
UNAIDS	35	46	68	90
**HIV testing (Adults)**				
IC2016	75	80	85	90
GOALS 2021	75	82	87	90
UNAIDS	75	82	87	90
**Community mobilisation**				
IC2016	25	35	43	50
GOALS 2021	28	3535	58	80
UNAIDS	28	-	58	80

MSM, men who have sex with men; FSW, female sex workers; UNAIDS, Joint United Nations Programme on HIV/AIDS; PrEP, pre-exposure prophylaxis; SRH, sexual reproductive health; SMC, safe male circumcisio.

## Appendix 2

**TABLE 1-A2:** Selected Outputs and Results for the 2016-IC, GOALS 2021, and UNAIDS Global AIDS Strategy for 2016, 2020, 2025, 2030.

Models	2016	2020	2025	2030
**Total PLWH population**				
IC2016	415 140	422 200	420 500	413 430
GOALS 2021	366 200	377 800	379 230	375 030
UNAIDS	-	-	376 980	369 950
**Total new HIV infections**				
IC2016	9642	4738	4140	3720
GOALS 2021	9070	5540	3800	3120
UNAIDS	-	-	2878	2770
**Incidence (adults 15+ years)**				
IC2016	0.75	0.35	0.28	0.24
GOALS 2021	0.80	0.45	0.27	0.20
UNAIDS	-	-	0.21	0.18
**Total deaths to the HIV population**				
IC2016	6711	5050	5200	5812
GOALS 2021	4700	4240	4440	4945
UNAIDS	-	-	4350	4810
**Total AIDS deaths**				
IC2016	4541	2589	1886	1510
GOALS 2021	2760	2250	1855	1610
UNAIDS	-	-	1780	1500

UNAIDS, Joint United Nations Programme on HIV/AIDS.

## Appendix 3: Data Sources For Spectrum V. 6.06 (2021)

**DemProj:**

- United Nation Statistics Division. 2020, World Population Prospects: The 2020 Revision. United Nations, New York, USA.

**AIM File:**

- MOHW, 2020. HAART Patient Update Summary, December 2020, DHAPC
- MOHW, 2020. Programme statistics: Breastfeeding, DHAPC, MOH.
- MOHW, 2020. Programme statistics: PMTCT coverage 2010–2020, DHAPC
- MOHW, 2020. Programme statistics on Knowledge of Status & Viral load suppression
- NACA BAIS, (II-2004, III-2008, IV-2013)
- NACA (2011) ANC Sentinel Surveillance Study

**GOALS and Resource Needs:**

- MOHW, 2020. Drug Costing and Forecasting Technical Working Group.
- MOHW, 2019. Mapping size estimation and behavioral and biological surveillance survey (BBSS) of HIV/STU among select high-risk sub-populations in Botswana. Technical Report, Botswana.
- Futures Group, 2013 One Health Tool. Futures Group.
- Charles Muthoga and Joseph N. Jarvis. Botswana Laboratory Costing. 2021

**Family Planning**

- Morronni, C. 2021, Sexual Reproductive Health Specialist, MOHW.

**Additional HIV & ART clinical and programmatic expertise provided by:**

- Ramaabya, D. 2021,MOHW: Head of HIV/AIDS Treatment, Care & Support
- Nkomo, B. 2021, MOHW: Head HIV/AIDS Programmes, Public Health Specialist
- Avalos, A. 2021, HIV Specialist Physician Botswana, Technical Advisor MOHW.

## Appendix 4

**TABLE 1-A4:** Numbers and percentage of MTCT Statistics GOALS-2021.

Category	Number	%
Started ART before pregnancy: child infected during pregnancy	26	11.4
Started ART during pregnancy: child infected during pregnancy	17	7.4
Dropped off ART during pregnancy: child infected during pregnancy	41	17.9
Started ART late in pregnancy: child infected during pregnancy	10	4.4
Mother infected during pregnancy (Not on ART)	38	16.6
Started ART before pregnancy: child infected during breastfeeding	2	0.9
Started ART during pregnancy: child infected during breastfeeding	1	0.4
Dropped off ART: child infected during breastfeeding	22	9.6
Mother infected during breastfeeding (Not on ART)	72	31.4

**Total**	**229**	**-**

MTCT, mother to child transmission.
